# Multisystemic Sarcoidosis Presenting With Leg Ulcers, Pancytopenia, and Polyserositis Was Successfully Treated With Glucocorticoids: A Case Report and Literature Review

**DOI:** 10.3389/fmed.2021.803852

**Published:** 2022-02-15

**Authors:** Xin Qiao, Shan He, Abdullah Altawil, Qiu-yue Wang, Jian Kang, Wen-yang Li, Yan Yin

**Affiliations:** Department of Pulmonary and Critical Care Medicine, First Affiliated Hospital of China Medical University, Shenyang, China

**Keywords:** splenomegaly, pancytopenia, sarcoidosis, glucocorticoid, case report

## Abstract

**Introduction:**

Sarcoidosis is a chronic granulomatous disease of unknown etiology. A variety of studies have pointed out that almost every part of the body can be affected, but it most often affected the lungs and intrathoracic lymph nodes. However, cases of sarcoidosis involving multiple organs in one patient are rarely reported. We describe a unique case of sarcoidosis, which was characterized by multiorgan involvement, including leg ulcers, splenomegaly, pancytopenia, and polyserositis. Glucocorticoids were effective during the treatment of the above lesions. This case highlights the diversity of clinical manifestations of sarcoidosis and emphasizes the importance of its differential diagnosis and the periodical follow-up. These are crucial to physicians in the diagnosis and treatment of sarcoidosis.

**Main Symptoms and Important Clinical Findings:**

A 30-year-old male complained about intermittent fever 3 years ago. A computed tomographic scan of the chest showed lymphadenopathy in the mediastinum and hilar regions. Routine blood tests showed leukopenia and mild anemia. The pathologic result of mediastinal lymph node biopsy was granulomatous lesions; thus, the patient was diagnosed with type II sarcoidosis without glucocorticoid therapy. In the following 2 years, the patient suffered from intermittent fever accompanied by dyspnea, fatigue, occasional cough, less sputum, and apparent weight loss. Abnormal physical examinations included leg ulcers and splenomegaly. Laboratory and physical tests revealed pancytopenia, polyserositis, and enlargement of lymph nodes. The pathological findings of leg ulceration, pleura, and left supraclavicular lymph node all suggested granulomas.

**Diagnosis, Interventions, and Outcomes:**

It strongly suggested sarcoidosis since tuberculosis, lymphoma, and connective tissue disease were all excluded. Due to severe conditions and multiorgan involvement, we tried to provide methylprednisolone for this patient. After 9 months of oral glucocorticoids therapy, his subjective symptoms as well as hematological and radiological findings were all improved. His leg skin ulceration and scab were also completely disappeared.

**Conclusion:**

Sarcoidosis has diverse clinical presentations, and many patients present with atypical symptoms. It needs to be timely identified by the clinician and carefully differentiated from other diseases with similar findings so as to make an accurate diagnosis. In this case, the patient had a poor clinical response to glucocorticoids in the early stage of treatment due to the severe condition and multi-organ involvement. It is worth noting that the patient had improved significantly after 9 months of treatment of corticosteroids, which suggested that follow-up is critical.

## Introduction

Sarcoidosis results from non-caseating granuloma formation due to ongoing inflammation, the latter of which causes the accumulation of activated T cells and macrophages. There are three criteria for the diagnosis of sarcoidosis: (1) a compatible clinical and radiologic presentation, (2) pathological evidence of non-caseating granulomas, and (3) exclusion of other diseases with similar findings such as infections (e.g., tuberculosis) and malignancy (e.g., lymphoma). Almost all organs can be affected by sarcoidosis, including the lung, eyes, lymph nodes, skin, spleen, liver, kidney, abdomen, heart, and bone marrow (1, 2). Monitoring extrapulmonary organs is essential because early recognition and treatment may prevent irreversible or life-threatening complications. Treatment with glucocorticoids should be considered for patients who have significant symptoms or are at progressive stage II or III of pulmonary disease or has a severe extrapulmonary disease. Second- and third-line therapies for pulmonary sarcoidosis, including immunosuppressants and biological agents, are reserved for patients with the corticosteroid-refractory disease, intolerable adverse effects, or corticosteroid toxicity (3).

To date, as far as we know, our case is extremely rare. First, the patient presented with splenomegaly, pancytopenia, and immune impairment, which were not clinically helpful to distinguish between benign and malignant. Second, massive pleural effusion and abdominal effusion were distinctly unusual in sarcoidosis. Third, the patient's liver and gallbladder may have been involved in sarcoidosis. Fourth, there was a poor clinical response to glucocorticoids at the early stage of treatment. Notably, long-term follow-up showed improvements in his symptoms, hematological deficits, polyserositis, and reduced nodule size up to 9 months after glucocorticoid treatment. The diagnosis of sarcoidosis was confirmed after the effective treatment response to glucocorticoids.

## Case Report

**Figure 1:**
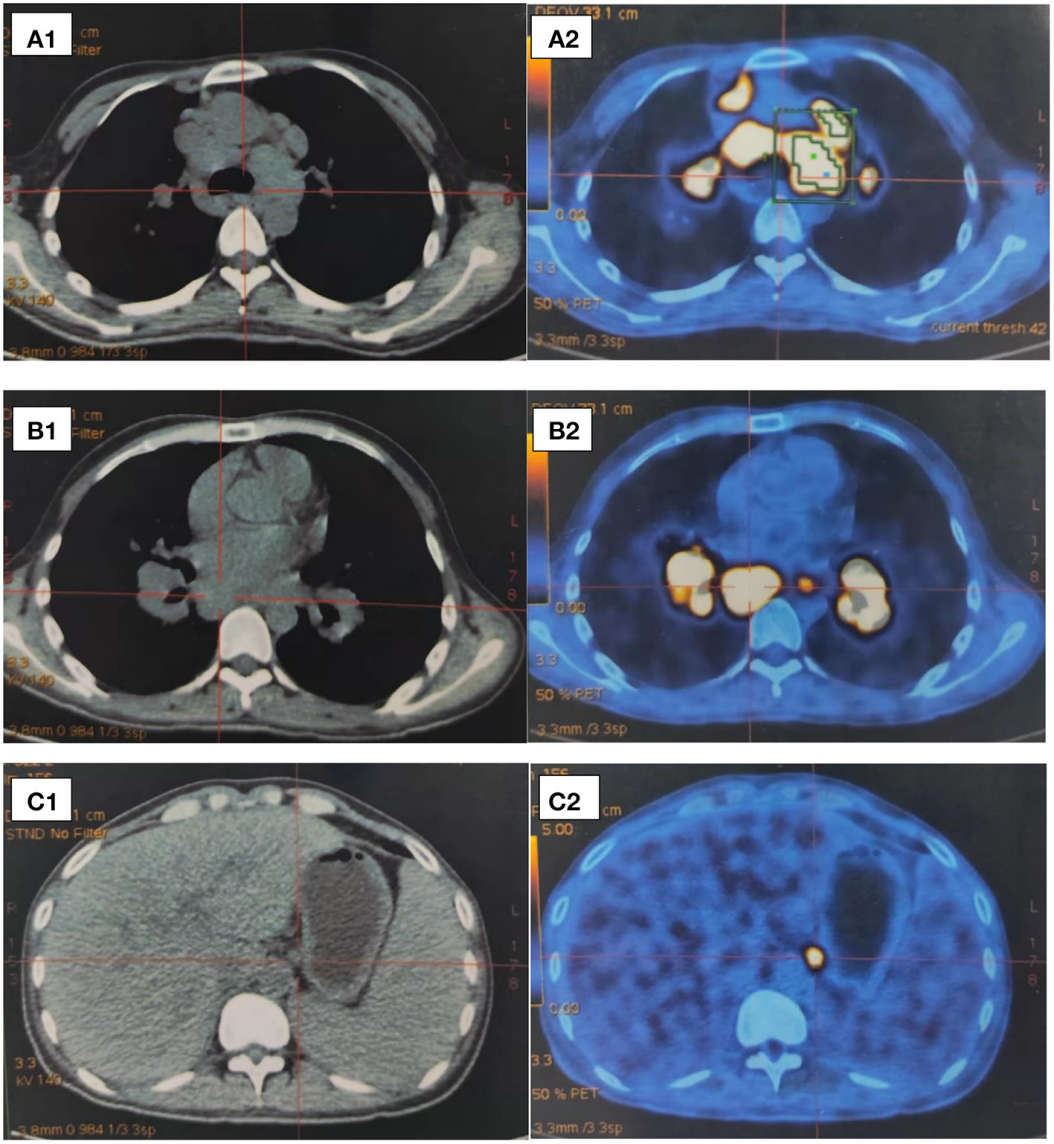
**(A–C)** Partial images of positron emission tomography-CT scan in 2017. **(A)** Mediastinal lymph nodes enlargement (tracheal carina) SUV max = 18.39; (aortopulmonary window) SUV max = 18.93. **(B)** Hilar lymph nodes enlargement. **(C)** Markedly enlarged spleen.

A 30-year-old male suffered an intermittent fever with a maximum temperature of 39°C since July 2017. He had a history of type 1 diabetes mellitus for 8 years. He was admitted to a local hospital and received cephalosporin therapy for a week, and then his temperature returned to normal. A CT scan of the chest showed lymphadenopathy in the mediastinum and hilar regions and multiple small nodules in both lungs. His white blood cell count was 2.9 × 10^9^/L with severe lymphopenia of 0.5 × 10^9^/L, and hemoglobin was 107 g/L, while the platelet count was average at 331 × 10^9^/L. He received a positron emission tomography-CT scan to identify whether it was benign or malignant, which revealed multiple enlarged lymph nodes with hypermetabolism in the bilateral hilum, mediastinum, axilla, and clavicle, and lymphoma could not be excluded (Figure 1). Subsequently, the patient underwent a biopsy of mediastinal lymph nodes performed by thoracoscopy, and the pathologic result was granulomatous lesions; thus, type II sarcoidosis was diagnosed. However, he did not receive glucocorticoid therapy with the consideration of his diabetes history and the early stage of sarcoidosis, and then he was told to recheck every 6 months. In the following 2 years, the patient suffered from intermittent fever (T max 37.7°C), which could be alleviated by oral antibiotics, accompanied by dyspnoea, fatigue, occasional cough, less sputum, and significant weight loss (lost 50 pounds). It is unfortunate that the patient was not regularly rechecked as prescribed. Last month, he complained that he had experienced progressive abdominal distention and dyspnoea, so he came to our hospital. The patient was sane without nausea and vomiting, and no positive signs were found on neurological examination. Abnormal physical findings consisted of a few scattered skin ulcers on the legs and splenomegaly extending 5 cm below the left costal margin. Abdominal ultrasonography revealed enhanced hepatic parenchyma echo, gallbladder (8.13 × 3.29 cm) and spleen enlargement (17.29 × 5.48 cm), and peritoneal effusion (depth of ~7.34 cm). An abdominal contrast-enhanced CT scan also revealed multiple retroperitoneal lymph nodes. A CT scan of the chest showed lymphadenopathy in the mediastinum and hilar regions with multiple nodules, patchy shadows, ground-glass opacities, and emerging bilateral pleural effusion as well as right atelectasis. Cardiac ultrasound reported that there were no abnormalities in cardiac structure, blood flow, or left ventricular systolic function, with an ejection fraction of 69%. No abnormal finding was seen in ECG. Ophthalmic tests including vision and fundus oculi were both normal. Superior lymph node ultrasound revealed enlargement throughout the left supraclavicular lymph nodes, bilateral inguinal nodes, popliteal lymph nodes, left neck lymph nodes, and bilateral axillary lymph nodes (Grade 3). Blood cell count was a remarkable abnormality with marked leucopenia and anemia. The liver function test suggested liver damage and hypoproteinemia. In addition, the patient also had severely impaired immune function. Laboratory test results are shown in Table 1.

**Table 1:** Laboratory test results and reference range.

	**Result**	**Reference range**
White blood cell	1.5 × 10^9^/L	(3.5–9.5) × 10^9^/L
Hemoglobin	91 g/L	(130–175) g/L
Platelet	144 × 10^9^/L	(125–350) × 10^9^/L
Calcium	1.99 mmol/L	(2.1–2.55) mmol/L
Albumin	27 g/L	(35–50) g/L
Aspartate transaminase	48 U/L	(15–46) U/L
Alanine transaminase	15 U/L	(13–69) U/L
Lactate dehydrogenase	999 U/L	(313–618) U/L
Gamma glutamate transpeptidase	198 U/L	(12–58) U/L
Alkaline phosphatase	793 U/L	(38–126) U/L
Brain natriuretic peptide	25 pg/ml	(0–100) pg/ml
Creatinine	51 umol/L	(58–110) umol/L
Urea	6 mmol/L	(3.2–7.1) mmol/L
C-reactive protein	19.4 mg/L	(0–6) mg/L
Erythrocyte sedimentation rate	40 mm/h	(0–15) mm/h
Mycodot	Negative	
T-SPOT	Negative	
Rheumatoid antibody	Negative	
Anti-nuclear antibody	Negative	
Anti-double stranded DNA antibody	Negative	
Anti-SM antibody	Negative	
Viral hepatitis serology	Negative	
Immunofixation electrophoresis	Negative	
CD4+ T cells count	82 cells/ul	(410–1590) cells/ul
Anti-neutrophil cytoplasmic antibodies	Negative	
Total bilirubin	23.2 umol/L	(3–22) umol/L

**Figure 2:**
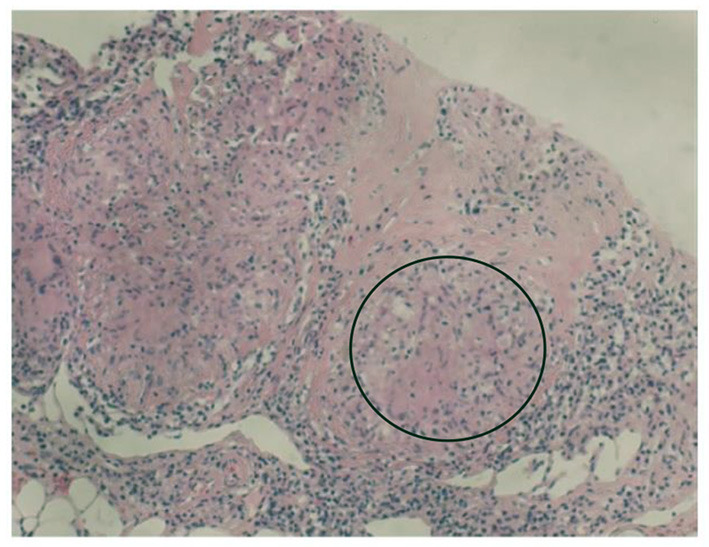
Biopsy of the left supraclavicular lymph node showing non-caseating granulomas (circle).

**Figure 3:**
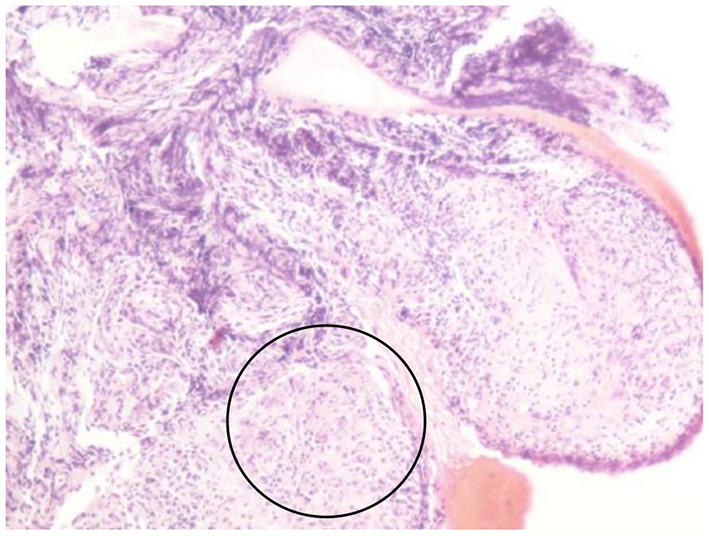
Pleural biopsy showing non-caseating granulomas (circle).

**Figure 4:**
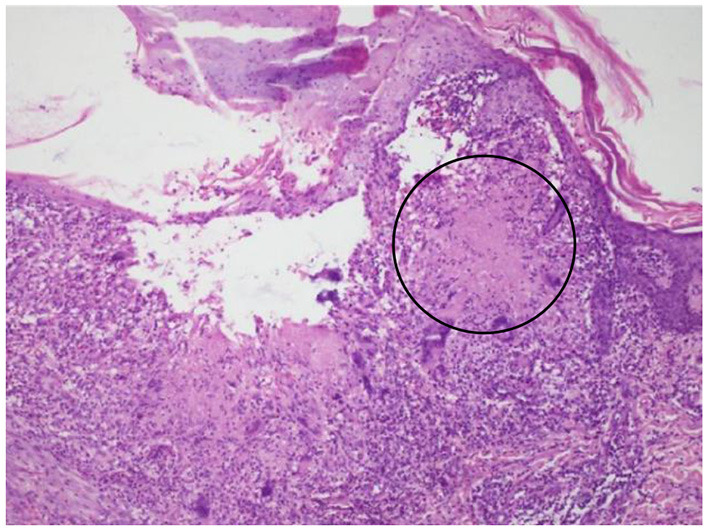
Biopsy taken from the left leg ulceration also showing granulomas (circle).

In summary, the patient had multiple organ involvement, and the disease progressed rapidly during these 2 years. During hospitalization, he took thoracentesis. As shown in Table 2, the pleural effusion was a yellow exudate. Cytopathologic examination of the pleural fluid was negative for malignant cells. In addition, both M. tuberculosis strains (acid-fast) and tuberculosis RT-PCR were negative. Subsequently, a biopsy of the left supraclavicular lymph node revealed the non-caseating granulomas with negative results for acid-fast staining and tuberculosis PCR. This result was in accordance with sarcoidosis (Figure 2). Histological analysis of the pleura obtained by thoracoscopy also revealed granulomas (Figure 3). A bone marrow aspirate and biopsy showed partial hypoplasia and no evidence of a lymphoproliferative disorder was found. In addition, a biopsy was taken from the left leg ulceration also revealed granulomas with a negative mycobacterium genetic test and culture results of fungi and bacteria (Figure 4). Abdominocentesis was not performed due to intestinal bloating and fewer ascites. Based on the above pathological results, the diagnosis of sarcoidosis was strongly suspected. Because of severe conditions and multiorgan involvement, 40 mg methylprednisolone was given intravenously once daily for seven consecutive days, followed by oral methylprednisolone 24 mg once daily.

**Table 2:** Pleural fluid routine tests.

**Test**	**Pleural fluid**	**Blood**
CEA (ug/L)	1	1.2
AFP (ug/L)	<0.91	1.13
CA12-5 (ug/L)	670	297
CA15-3 (ug/L)	23.1	34.1
CA19-9 (ug/L)	16.3	44.1
LDH (U/L)	250	405
TP (g/L)	43.1	60.7
ADA (U/L)	33.71	
Glu (mmol/L)	7.78	
Total cell count (× 10^6^)	288	
White blood cell count (× 10^6^)	279	
Red blood cell count (× 10^6^)	0.012	
Mononuclear cell count (× 10^6^)	273	
Ratio of mononuclear cells (%)	98	
Multinuclear cell count (× 10^6^)	6	
Ratio of multinuclear cells (%)	2	
Rivalta test	**+**	

*CEA, carcinoembryonic antigen; AFP, alpha-fetoprotein; CA, carbohydrate antigen; LDH, lactate dehydrogenase; TP, total protein; ADA, adenosine deaminase; Glu, glucose*.

**Figure 5:**
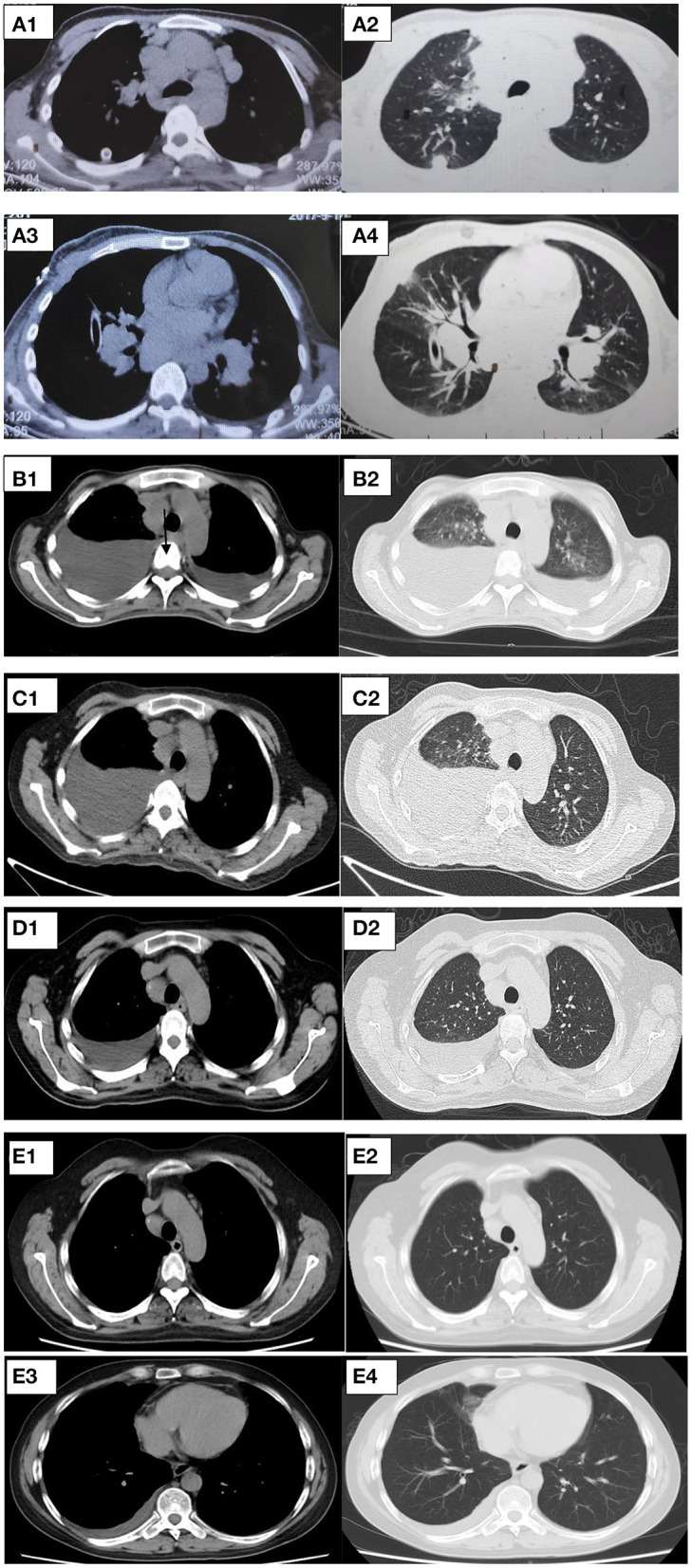
**(A–D)** Representative CT cuts of the thorax during the course of the disease. **(A)** Chest CT in 2017. **(B)** Pre-glucocorticoid therapy (September 29, 2020, chest CT). **(C)** One month after glucocorticoid therapy (November 25, 2020, HRCT). **(D)** Six months after glucocorticoid therapy (March 20, 2021, HRCT). **(E)** Nine months after glucocorticoid therapy (June 30, 2021, chest contrast-enhanced CT). CT, computed tomography; HRCT, high resolution computed tomography.

**Figure 6:**
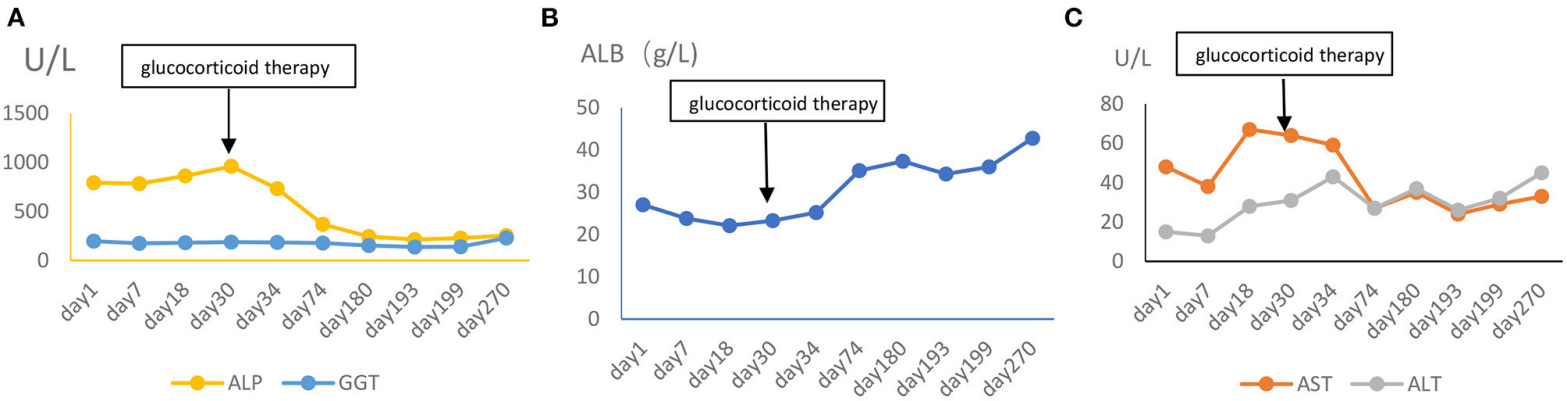
**(A–C)** The change of hepatic function (ALP, alkaline phosphatase; GGT, gamma glutamate transpeptidase; ALB, albumin; ALT, alanine transaminase; AST, aspartate transaminase).

**Figure 7:**
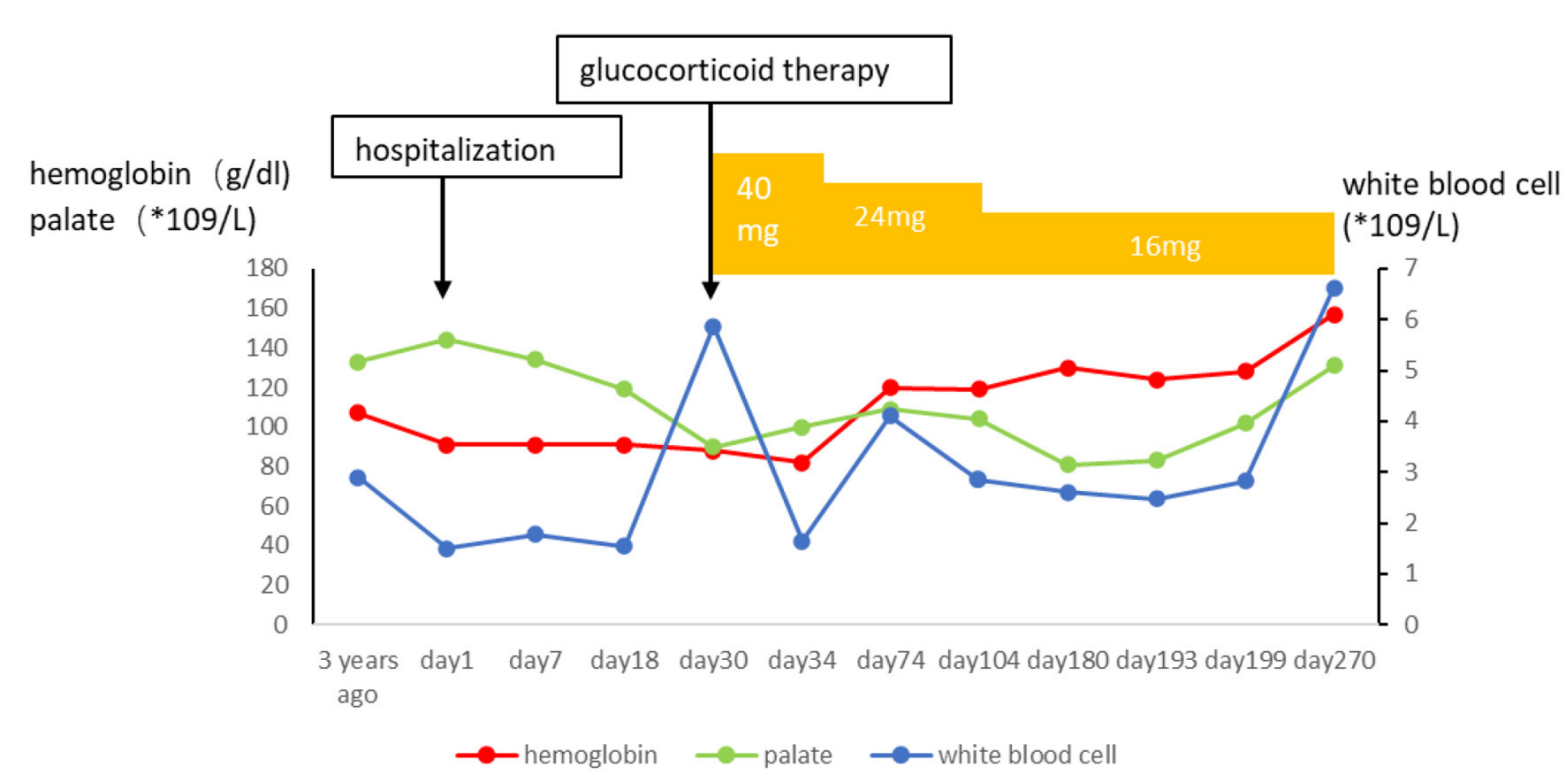
Effects of glucocorticoid on hematological parameter.

**Figure 8:**
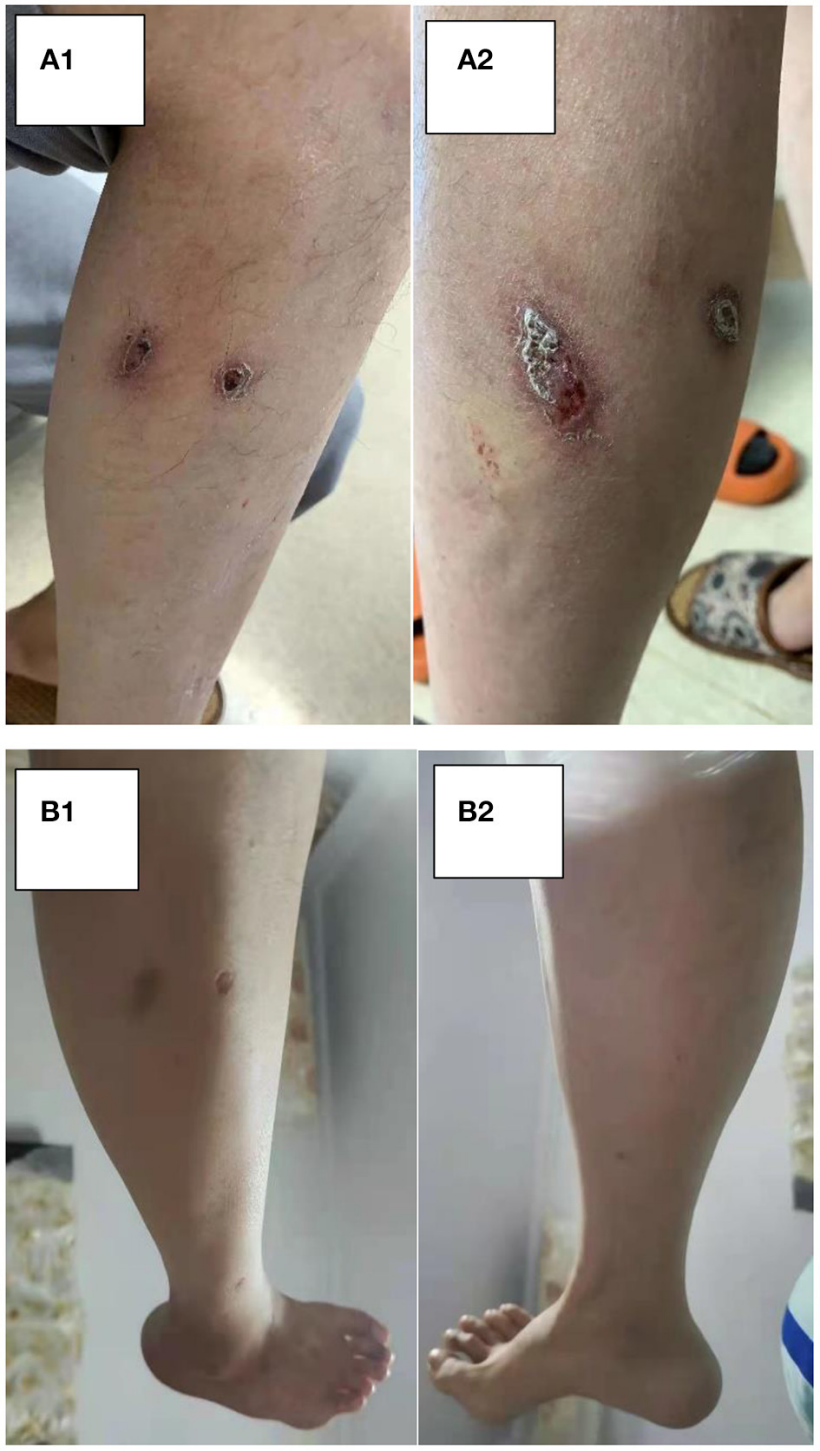
**(A,B)** The change in leg skin. **(A)** Pre-glucocorticoid therapy (September 13, 2020). **(B)** Nine months after glucocorticoid therapy (June 30, 2021).

After 1 month of methylprednisolone therapy for 24 mg once daily, there was no reduction in pleural effusion on high-resolution CT (HRCT), and the spleen was larger than before; thus, we continued to maintain the current dosage (24 mg once daily). After another months' treatment, the right pleural effusion was decreased slightly, then the dose of methylprednisolone was reduced to 16 mg once daily. Six months later, a follow-up HRCT showed that the bilateral pleural effusion was significantly less than before (Figure 5). Hepatic function (Figure 6) and superficial lymph nodes and spleen were reduced. After methylprednisolone administration (the dosage was still 16 mg/day) for 9 months, the patient became asymptomatic with a normal blood cell count (Figure 7), and the CD4+ T cell count increased from 82 to 162. Ascites were negative, and the spleen and gallbladder were both reduced. In addition, the leg skin ulcers and scabs completely disappeared (Figure 8). We then focused on the patient's side effects of glucocorticoids. The patient had no psychiatric symptoms or gastrointestinal discomfort. Blood glucose levels were reasonably controlled by subcutaneous injection of insulin (fasting blood glucose and postprandial blood glucose levels were maintained at 6–8 and 8–10 mmol/l, respectively).

## Discussion and Conclusion

The strength of our study is that it is the first case of sarcoidosis with systematic manifestations, including skin, spleen, pleura, lymph node, and abdomen manifestations, which were probably sensitive to glucocorticoid therapy. However, our study also had a limitation. We lack pathological evidence of abdominal organ involvement, such as the peritoneum, spleen, liver, and bladder. Furthermore, there was no significant improvement in splenomegaly, which may be related to the insufficient courses of treatment or the overlapping presentations. Glucocorticoids have been reported to be the first-line treatment for sarcoidosis. But our patient is at high risk of side effects with glucocorticoid therapy because of diabetes history. Conventional and biological immunosuppressive drugs such as methotrexate and tocilizumab have been studied for their glucocorticoid-sparing properties to reduce glucocorticoid exposure and maintain remission (4). Therefore, we may first consider glucocorticoid-sparing therapy in patients with high-risk factors for glucocorticoid complications. Finally, given the patient's splenomegaly, systemic lymphadenopathy, and reduced immune function, although there is currently no pathological support for the diagnosis of lymphoma, it can occur 2–8 years after the sarcoidosis diagnosis most of the time, preferentially in patients with a chronic course of the disease (5). The patient requires a more extended period of treatment and consequent follow-up clinic.

Pulmonary involvement can be seen in more than 90% of patients with sarcoidosis. Almost all organs can be affected by sarcoidoses, such as the spleen, liver, kidney, abdomen, heart, and bone marrow. However, the eyes, lymph nodes, and skin are the most commonly involved organs (6–8). Our study provides a systematic literature review of the patient's several unusual extrapulmonary manifestations in the following report.

Cutaneous involvement occurs in nearly one-third of sarcoidosis patients (9), and the lesions can exhibit many different manifestations, which are divided into two categories: “specific,” where histological examination shows typical non-caseating granulomas, and “non-specific,” where there is a lack of the former feature (10). The specific lesions include lupus pernio, infiltrated plaques, maculopapular eruptions, subcutaneous nodules, and scars, whereas a variety of unusual forms include erythrodermic, ichthyosiform, psoriasiform, verrucous, or ulcerated sarcoidosis (11). Although ulcerative sarcoidosis remains rare, it has been increasingly reported in the last few decades. A previous review (12) of 147 patients with cutaneous sarcoidosis found ulceration in 4.8% of patients, which is consistent with Powell's study (13). We reviewed 17 cases with full text from the English-language literature based on a PubMed search using the terms ulcerative sarcoidosis and case report for the last 20 years (13–28). They are presented in Table 3. We found a female to male ratio of 2.4–1, and the average age of presentation was 52 years (age range, 26–73 years). Approximately 76% (13/17) of cases presented with involvement of the legs. Additionally, ulcers were generally solitary or sporadic lesions with necrotic yellow, blue, or red plaques accompanied by a foul smell and discharge. Upon histologic examination of ulcerative sarcoidosis, 11 of the ulcerative sarcoidosis in our review were described as either epithelioid granulomas or non-caseating granulomas. However, several cases also showed atypical features, including caseation necrosis and granulomatous vasculitis, including our case, which did not generally seem to be compatible with sarcoidosis. The histologic difference in these cases may primarily include an infection aspect. Moreover, extensive workup needs to be done in these patients to ensure that it was not only an infection. The lesions should also be distinguished from other granulomatous disorders, such as granulomatosis with polyangiitis (GPA), eosinophilic granulomatosis with polyangiitis (EGPA), and lymphomatous granulomatosis (29). In terms of laboratory tests, only five patients presented with elevated ACE, while one declined in our review. Although elevated ACE levels have been seen in patients with sarcoidosis, the ACE level lacks specificity, and it has large interindividual variability, which limits its clinical utility (30).

**Table 3:** Reported cases of ulcerative cutaneous sarcoidosis for recent 20 years.

**References**	**Patient A/S**	**Description**	**Location**	**Laboratory tests**	**Sarcoid elsewhere**	**Ulcer pathological finding**	**Treatment and outcome**
1. Powell and Rosen (13)	49/M	Ulcerations with foul-smelling, greenish yellow, purulent drain-age	Legs	Bacterial and fungal and TB (–)	Not reported	Non-necrotizing granulomatous inflammation with many Langerhans-type giant cells	Prednisone plus hydroxychloro-quine, 3 years without relapse
2. Vetos et al. (14)	60/F	ulcerative necrobiosis lipidic-like plaques	left arm	ACE: normal Fungi and TB (-)	NO	Naked sarcoid granulomas with minimal lymphocytic infiltrate and granulomatous vasculitis	Adalimumab, 4 months improvement
							Non-effective: hydroxychloroquine, methotrexate, sulfasalazine, antibiotics
3. Shatnawi et al. (15)	44/F	Ulcerative form of pyoderma gangrenosum	Legs	Not reported	Yes: lymph node	Non-caseating granuloma with abscess formation	Split-thickness skin grafting without immunosuppressive treatment within 4 months, improvement
4. Chaabani et al. (16)	57/F	Multiple infiltrated blue-red plaques with an ulcerated and atrophic center	Face, trunk, buttocks, and limbs	PPD (–), elevated ACE	Not reported	Multiple non-caseating granulomas	Oral prednisone and hydroxychloroquine with topical betamethasone, slight improvement, lack of follow-up
5. Bukiej et al. (17)	39/M	Ulcerative lesions with foul smelling purulent drainage	Legs	Elevated ACE, Fungi and TB (–)	Yes: lymph nodules, spleen	Non-caseating granulomas	Infliximab, hydroxychloroquine, oral prednisone, 5 months improvement
6. Noiles et al. (18)	47/F	Ulcers with serous discharge	Left leg	*Escherichia coli* (+) TB (–), fungi (–), PPD (–), normal ACE	Yes: lungs, liver, and spleen	Necrotizing granulomatous inflammation with a lymphocytic infiltrate	Infliximab, prednisone, cyclosporine, dapsone few months, methotrexate or infliximab -induced injury, pass away
	62/F	Extensive infiltrated eroded and ulcerated red-brown and violaceous plaques	Left arm	*Staphylococcus aureus* (+)	Yes: lymph node	Classic sarcoid granulomas as well as varying degrees of necrotizing granulomatous inflammation	Cyclosporine, prednisone and methotrexate, improvement
7. Ichiki and Kitajima et al. (19)	59/M	Linear ulcers and reticulated reddish-blue discoloration	Left leg	Elevated ACE PPD (–), TB (–)	Yes: Lung, lymph nodes	Caseation necrosis and Langerhans' giant cells were partially seen among the epithelioid granulomas	Prednisolone, 6 months, improvement
8. Poonawalla et al. (20)	45/F	Punched-out ulcerations	Both lower legs	Normal ACE, Fungi and TB (-)	Yes: lymph nodes	Non-necrotizing granulomas with focal involvement of medium-sized blood vessels with areas of vessel-wall damage (Granulomatous vasculitis)	Prednisone and azathioprine,3 months, improvement
9. Fujii and Torii et al. (21)	68/F	Violaceous plaques accompanied by ulceration with moth-eaten appearance	Right knee	TB (–), fungi (–), bacteria (–), elevated ACE	Yes: lung, lymph nodes	Non-caseating epithelioid cell granulomas consisting of epithelioid histiocytes and giant cells	Topical corticosteroid, 20 days, improvement
10. Wollina et al. (22)	45/F	Pretibial ulcers	Legs	*Staphylococcus epidermidis* (+), elevated ACE	Yes: lymph node, lung, spleen	No sarcoid granulomas right groin disclosed naked granulomas	Oral prednisolone, Topical fluocinolone-neomycin ointment, 6 months, improvement
11. Barisani et al. (23)	64/F	Ulcers	Bilateral pretibial region	Declined ACE, Staphylococcus aureus (+), TB (–)	No	Naked granulomas with epithelioid and giant cells	Methylprednisolone, a few days improvement
12. Wei et al. (24)	26/M	Multiple purpuric ulcers	Lower limbs	Pathogen (–)	Yes: testes, lymph nodes	Non-caseating granulomas and multinucleated giant cells surrounding blood vessels with nuclear dust and extravasated red blood cells (Granulomatous vasculitis)	Prednisolone plus azathioprine, 5 months, improvement
13. Philips et al. (25)	55/F	Ulcer measuring 9.8 cm *3.8 cm	Right lower extremity	Tissue cultures (-)	Not reported	Cutaneous sarcoidosis	Persisting despite treatment with prednisone, hydroxychloroquine, and methotrexate, improvement: 9 weeks of treatment with adalimumab.
14. Streit et al. (26)	73/F	Atrophic, dry and scaly with ulcers, erosions and crusts,10 cm	Shins	Slightly elevated ACE	No	Aggregates of epithelioid cells with multinucleate giant cells without caseation under the epidermis	Steroid with triclosan ulcer enlargement, Apligraf, 6 months, improvement
15. Hashemi and Rosenbach (27)	50/F	A 10–12 cm shallow ulcer, the ulcer surface had a thick, yellow, foul-smelling adherent crust	Scalp	Unremarkable	Yes: lung	Granulomatous inflammation in the dermis (nodular aggregates of epithelioid histiocytes multinucleated giant cells with asteroid bodies and a surrounding lymphoplasmacytic infiltrate)	Mild improvement after treatment with hydroxychloroquine followed by more significant improvement with treatment of weekly adalimumab injections
16. Kluger et al. (28)	36/M	Extensive, irregular, geographic, and serpiginous ulcers, bases were covered with yellowish and hemorrhagic sloughs	Anterior aspect of both legs	Mild leukopenia, thrombopenia, fungal and TB (-)	Yes: mediastinal and abdominal lymph nodes, liver, spleen	Epithelioid and histiocytoid granulomas with small area of necrosis without caseation	Oral prednisolone, improvement

Corticosteroids have been reported to improve the symptoms of sarcoidosis in all organs. Our patient showed a good response to methylprednisolone therapy for his skin ulcers. In our review of ulcerative sarcoidosis, many types of treatments have been reported, such as skin grafts, antimalarials (chloroquine or hydroxychloroquine), topical corticosteroids, anti-ulcer cream, immunosuppressive drugs (azathioprine or cyclosporine), and biologic agents (adalimumab or infliximab). However, failures and side effects were also accompanied by the above treatments, including corticosteroid-induced hyperglycaemia and methotrexate- or infliximab-induced organ injury. Therefore, it is essential for us to pay attention to the response and the side effects of drug treatment depending on the patients' conditions.

Previous studies have reported that splenomegaly was present in 5.6–50.0% of sarcoidosis cases (31, 32). We described 22 cases presenting splenomegaly and sarcoidosis with histological proof in the last 20 years, as shown in Table 4 (33–53). In the spleen, massive splenomegaly is the most common presentation, followed by multiple splenic lesions (54). Massive splenomegaly [defined when it extends into the pelvis, when it has crossed the midline of the abdomen, or when it has a weight over 1,000-1,500 g or the largest dimension >20 cm (42)] was clinically perceptible in 12 patients. There were six patients who presented with an in-homogeneously enlarged spleen with hypoechoic nodular lesions. The more frequent clinical features were digestive symptoms (including abdominal distention, appetite loss, nausea, vomiting, early satiety, dyspepsia, abdominal pain in 13 cases) and constitutional symptoms (fever *N* = 3; fatigue *N* = 7; weight loss *N* = 9). Serum angiotensin-converting enzyme (ACE) and serum calcium levels were elevated in 68 and 23% of cases, respectively. In addition, sIL-2R and liver enzymes were elevated in several patients. Therefore, measurement of the above marker levels, even though not diagnostic, may be helpful to monitor activities and evaluate the therapeutic effect. Moreover, of the 23 cases, including our patient, 16 cases had haematologic abnormalities (pancytopenia *N* = 8; bicytopenia *N* = 5; solely anemia *N* = 3). Several putative mechanisms may explain haematologic abnormalities in sarcoidosis: hypersplenism, bone marrow involvement, and autoimmune destruction, such as immune thrombocytopenia (ITP) and lymphoma (55), which represent a diagnostic challenge. Therefore, we emphasize that biopsies of bone marrow, spleen, and lymph nodes may be necessary to identify the cause. Pancytopenia was presented in two patients with bone marrow involvement, and thrombocytopenia was present in one patient with ITP. It is worth mentioning that a case of splenomegaly and abnormal blood cell counts were secondary to bone marrow infection with Leishmania protozoa, which needs our attention to exclude parasitic infection. The coexistence of sarcoidosis and opportunistic infection, even in the absence of any immunosuppressive therapy, has previously been documented (56).

**Table 4:** Cases of sarcoidosis with splenomegaly in the last 20 years.

**References**	**Patient A/S**	**Clinical presentation**	**Laboratory tests**	**Sarcoid involvement**	**Pathological finding**	**Treatment and outcome**
1. Saito et al. (33)	22/W	Abdominal distention, fatigue, and appetite loss, weight loss, massive splenomegaly (28 × 21 cm) 4300 g	Pancytopenia, liver disturbance, elevated sIL-2R, ACE, lysozymes, KL-6	Lymph node, lung, liver, spleen, skin	Spleen: epithelial granuloma with multinucleated giant cells, normal bone marrow biopsy	Splenectomy to improve pancytopenia (platelet count increased, ACE, lysozymes, and sIL-2R decreased slightly, pulmonary symptoms disappeared) later consider steroid treatment because of incomplete improvement in other organs
2. Stoelting et al. (34)	58/F/black	Nausea, vomiting, early satiety, weight loss, massive splenomegaly (28*20*12) 2,875 g, multiple splenic artery aneurysms	Anemia, thrombocytopenia	Spleen	Spleen: non-caseating granulomas, normal bone marrow biopsy	Partial splenic artery embolization control bleeding and normalizing the platelet count, diagnostic splenectomy
3. Akaba et al. (35)	23/F/Japanese	Massive splenomegaly (21 × 15 × 10 cm), abdominal distention	Elevated ACE, lysozyme, sIL-2R; decreased WBC and palate	Liver, lung, spleen	Liver, spleen and lung: non-caseating granuloma	Splenectomy because of progressive cytopenia and high risk of splenic rupture (ACE and lysozyme, and abdominal distention improved after splenectomy)
4. Kawano et al. (36)	58/F	Weight loss, massive splenomegaly (21 × 15 × 10 cm)	Elevated sIL-2R and ACE, Slight anemia	Heart, lymph node, skin, eye, liver, spleen	Skin: erythema nodosum epithelioid granuloma with multinucleated giant cells of Langerhans and no caseous necrosis spleen: epithelioid granuloma with multinucleated giant cells of Langerhans, asteroid body, and no caseous necrosis	Diagnostic splenectomy to exclude lymphoma, prednisolone to improve the cardiac and ocular lesion
5. Giovinale et al. (37)	53/F	Epigastric repletion, splenomegaly (13 × 7 × 7 cm, 240 g) with hypoechogenic nodular lesion	Normal	Lymph nodes, spleen	Spleen: non-caseating granulomas	Splenectomy to exclude lymphoma, no further treatment
	32/F	Epigastric repletion, enlarged spleen with numerous round hypoechogenic nodules, 280 g,15 × 7.4 × 6 cm	Elevated ACE	Liver, lymph nodes, spleen	Spleen: chronic noncaseating epithelioid cell granulomas with Langerhans multinucleated giant	Diagnostic splenectomy, no further corticosteroids treatment
6. Akinsanya et al. (38)	20/M	Massive splenomegaly (30 × 18 × 8 cm)1800 g, dyspepsia, fatigue	Pancytopenia, elevated ACE and ALP, fungus and TB (-)	Liver, spleen	Spleen: non-caseating epithelioid cell granulomas	Splenectomy based on increasing fatigue, abdominal discomfort on the account of splenomegaly, and progressive pancytopenia., 3 days
					Liver: granulomatous, normal bone marrow biopsy cells	Later, all blood cells are elevated
7. Palade et al. (39)	66/F	Massive splenomegaly III-IV (lower pole is below the navel 25/15/9 cm)	Anemia	Spleen	Spleen: numerous sarcoid granulomas type with-out caseating necrosis	Splenectomy: hematological improvement
8. Bachmeyer et al. (40)	56/F	Weight loss and abdominal pain, massive nodular splenomegaly (24 cm in length, extended to the pelvis)	Anemia, leucopenia, elevated ACE	Labial salivary gland	Labial salivary gland biopsy revealed typical sarcoid granuloma and the absence of central necrosis, normal bone marrow biopsy	Prednisolone, improvement within 3 months of the s, splenomegaly and hematological abnormalities
9. Saba et al. (41)	72/F	Anorexia fatigue, massive splenomegaly (23 cm in length)	Hypercalcemia, Pancytopenia, elevated ACE	Bone marrow	Bone marrow biopsy showing hypercellularity and a non-necrotizing granuloma	Prednisone, calcium level normalized (no follow-up)
10. Paul et al. (42)	65/M	Fatigue, lack of appetite, weight loss, massive splenomegaly (20.7 cm)	Hypercalcemia, pancytopenia, elevated ACE, impaired liver function	Bone marrow, liver	Liver and bone marrow biopsy: non-necrotizing granulomas	Not reported
11. Haran et al. (43)	53/F	Massive splenomegaly (1600 g, 15 cm below the left costal margin, 20 cm span)	Pancytopenia, elevated ACE	Liver, spleen	Spleen: multiple non-caseating granulomas, normal bone marrow examination	Diagnostic splenectomy to exclude splenic lymphoma, complete resolution of her cytopenia, no further treatment
12. Ravaglia et al. (44)	42/F	Fever, fatigue, hepatomegaly and splenomegaly.	Lymphopenia, anemia	Lymph node, lung	Had been diagnosed sarcoidosis, Bone marrow biopsy containing leishmania protozoa	Splenomegaly and Lymphopenia second to visceral leishmaniosis, given liposomal amphotericin, lymphocytes increased later
13. Sreelesh et al. (45)	50/F	Loss of appetite and weight loss, abdominal discomfort and early satiety. Splenomegaly(210 g) with multiple hypoechoic lesions	No	Spleen	Spleen: non-caseating granuloma composed of epithelioid histiocytosis, multinucleated giant cells	Diagnostic splenectomy remained asymptomatic 3 years
14. Jhaveri et al. (46)	40/F	Fever, fatigue, night sweats, enlarged spleen measuring 16 × 7 × 6 cm with multiple hypodense lesions	Elevated ACE and leukocyte count	Spleen	Spleen: multiple noncaseating granulomas with multiple histiocyte-consisting follicles, normal bone marrow examination	Diagnostic splenectomy, over the next 3 months asymptomatic, ACE normal
15. Mohan et al. (47)	39/F	Fever, anorexia, malaise, and weight loss, massive splenomegaly (22 cm below the costal margins) with multiple hypodense lesions	Elevated ACE, Pancytopenia	Cervical lymph node, skin	Skin: non-caseating epithelioid granulomas; cervical lymph node: non-necrotizing epithelioid cell granulomas normal bone marrow biopsy	Oral prednisolone hematologic parameters improved reduction in lymph nodes and spleen with disappearance of the low attenuation lesions
16. Mattia et al. (48)	12/F	Asthenia and weight loss, palpable spleen	Mild anemia	Lung, lymph node, liver	Liver and lymph node: non-necrotizing granulomatous inflammation	Prednisone, without other clinical signs or symptoms, normal laboratory tests
17. Xiao et al. (49)	43/F	Massive splenomegaly (1.82 kg, 21 cm extend to the level of the umbilicus and across the midline.)	Increased calcium and ACE	Spleen, lymph nodes, skin	Spleen and peri-splenic lymph nodes biopsy: epithelioid granuloma	Splenectomy to exclude lymphoma
18. Medhat et al. (50)	38/M	Gingival bleeding, and epistaxis, splenomegaly	Severe thrombocytopenia, anemia,	Lymph nodes, lung	Lymph nodes and lung: non-caseating granuloma. Bone marrow aspirate and biopsy: immune thrombocytopenic purpura (ITP)	Platelet transfusion, methylprednisolone, immunoglobulin G and romiplostim, sustained normalization of platelet count
19. Sherief et al. (51)	9/F	Abdominal pain and anorexia, Splenomegaly 19.5 cm	Pancytopenia, slightly higher ACE,	Lymph nodes, spleen	Lymph node and spleen: non-caseating granulomas, normal bone marrow biopsy	Splenectomy due to hypersplenism, blood counts returned to normal. After 2 years, multiple lymph nodes enlargement, given prednisolone, later clinical remission
20. Morton (52)	28/M	Vomiting and weight loss, hepatosplenomegaly, multiple lymphadenopathy	Hypercalcemia, elevated ACE	Lung, lymph nodes	Lung biopsy: non-necrotizing granulomas	Methotrexate and Prednisolone, resolution of pulmonary infiltrates, normal serum calcium
21. Barwell and Peden (53)	67/M	Multiple subcutaneous skin nodules, splenomegaly	Elevated calcium and ACE	Lymph nodes, lung, skin	Skin: naked granulomata	Prednisolone, skin lesions fully regressed and his biochemistry had normalized

No treatment is required when splenomegaly is asymptomatic. When disabling, treatment relies on oral corticosteroids, which were also highly effective in our patients. In our review, hematological abnormalities improved after splenectomy or glucocorticoid therapy in six and three patients, respectively. Splenectomy is necessary for patients who fail to respond to pharmacotherapy, massive splenomegaly with pressure-related symptoms, prophylaxis for splenic rupture, treatment for refractory cytopenia, or diagnostic exclusion of malignancy, especially splenic marginal zone lymphoma (42, 54). However, splenectomy could develop life-threatening infection postoperatively, and patients are at an increased risk of vascular complications, including pulmonary embolism, deep vein thrombosis, and portal and splenic vein thrombosis (57). The exacerbation of DPB has been reported in the literature after splenectomy (35). Likewise, the risks of glucocorticoid therapy also need to be weighed against the potential benefits.

Another feature of this patient's illness was bilateral pleural effusions and ascites, which have been thought to be unusual in sarcoidosis. In a study of 181 outpatients with sarcoidosis, 2.8% had pleural effusions, and only 1.1% had sarcoid pleural involvement (58). The mechanism of pleural effusion formation in sarcoidosis could be attributed to the following five points: (1) pleural involvement, (2) superior vena cava obstruction (59), (3) bronchial stenosis and lobar atelectasis resulting from endobronchial sarcoidosis (60), (4) trapped lung (61), and (5) chylothorax leading to lymphatic disruption (62). Ascites are also rare manifestations of sarcoidosis. It may appear as a result of portal hypertension-related to liver involvement or severe pulmonary involvement with a serum ascites albumin gradient (SAAG) higher than 1.1 g/dl and peritoneal involvement with a SAAG lower than 1.1 g/dl. Very few cases presented both pleural effusion and ascites (63). As shown in Table 5, of the 22 patients with pleural effusion or ascites from 2010 to 2021 (64–82), there was an increased incidence in women (14/22), 17 (77%) had pleural effusion, two (9%) patients had ascites and three (17%) patients had both. In 21 cases with pleural effusion, including our case, 10 cases had biopsy-proven pleural involvement. Sarcoid pleural effusions are more commonly left-sided (48%, 10/21) and less frequently bilateral (24%, 5/21), and they are often exudative and lymphocytic. The etiology of pleural effusion in sarcoidosis requires us to rule out some possible diseases, such as connective tissue diseases, tuberculosis, carcinoma, lymphoma, and chronic heart failure. In our patient, we performed a detailed differential diagnosis. Given that pleural effusion was an exudate and there were no abnormalities in brain sodium peptide, renal function, or rheumatism-related antibodies, we could temporarily rule out heart failure hypoproteinemia, nephrotic syndrome, and rheumatic disease (such as lupus). Moreover, acid-fast staining, tuberculosis PCR, and malignant cells were all negative in pleural fluid. The most powerful point was that the histopathology of pleural demonstrated granulomas consistent with sarcoidosis. Similarly, in six cases with ascites, including our case, one case had peritoneal involvement, one case had gastrointestinal involvement, and one case was because of portal hypertension. The most commonly reported manifestations were abdominal pain, abdominal distention, nausea, vomiting, and fever. The most commonly reported manifestations were colicky abdominal pain, chills, and feverishness. Several diseases should routinely be investigated and ruled out in patients with ascites, including inflammatory, infectious, and neoplastic diseases. An abdominal laparotomy or laparoscopy is sometimes required to reveal the involvement of granulomatous disease in the viscera and peritoneum. In addition, elevated ACE levels were seen in almost 73% (16/22) of cases. CA-125 can also be elevated in three cases, and one patient had normalization of CA-125 levels after treatment with prednisolone. However, the diagnostic significance of these biomarkers in sarcoidosis remains uncertain.

**Table 5:** Cases of sarcoidosis with pleural effusion or ascites from 2010 to 2021.

**References**	**Patient A/S**	**Presentation**	**Sarcoid involvement**	**Laboratory tests**	**Pleural effusion**	**Ascites**	**Pathological finding**	**Treatment and outcome**
1. Gunasekharan et al. (64)	63/M	Recurrent pleural effusions, back pain, weight loss	Bone marrow, lymph nodes	Elevated PTHrP, hypercalcemia	Yes: left-sided, lymphocytic exudate with blood	No	Bone marrow: non-caseating granuloma	Prednisone, a complete resolution of symptoms and decreased pleural effusion
2. Lee et al. (65)	32/F	Abdominal discomfort, weight loss	Lymph node	Elevated ACE	Yes: left unilateral pleural effusion	Yes, peritoneal thickening	Lymph node: capsular fibrosis and numerous granulomas	Corticosteroid, decreases in the numbers and sizes of enlarged lymph nodes, and improvement in the ascites and peritoneal thickening.
3. Rivera et al. (66)	65/F	Shortness of breath, hypoxia, and cough	Lymph node and pleura	Normal	Yes: large right, lymphocyte predominant transudate without blood	No	Mediastinum lymph node and pleura: noncaseating granulomas	Prednisone, PE improved
4. Ferreiro et al. (67)	45/M	Chest pain and dyspnea	Lymph node	Elevated ACE	Yes: right	No	Hilar lymph node: non-necrotizing epithelioid granulomas	Corticosteroids, PE resolved
	83/F	Pleuritic pain and dyspnea	Lymph node	Elevated CA-125	Yes: left	No	Hilar lymph node: non-necrotizing granulomas	Corticosteroids, resolution of PE
	39/M	Asthenia, cough, dyspnea	Hilar lymph node	Elevated ACE	Yes: left	No	Hilar lymph node: non-necrotizing granulomatous inflammation	PE resolved after treatment with corticosteroids.
5. Joshi et al. (68)	42/M	Shortness of breath, loss of appetite and weight-loss	mediastinal lymph node, lung, pleura	Elevated ACE, PPD (-)	Yes: left, exudative lymphocytic fluid	No	Pleural biopsy: epithelioid granulomas without necrosis	Prednisolone, Hydroxy chloroquine (Pleural effusion appears after a period of oral prednisolone
6. Mota et al. (69)	44/F	Epigastric and periumbilical pain, vomiting, abdominal distension, asthenia, and anorexia.	Gastrointestinal tract	Anemia, elevated ACE, hypercalcemia	Yes: left	Yes: mild	Gastric and colon biopsies: epithelioid granulomas without necrosis	Methylprednisolone, significant improvement of ascites and pulmonary nodules, pleural effusion, adenopathy as well as gastric lesions
7. Hou et al. (70)	49/F	Chest tightness, fatigue and dyspnea	Hila and mediastinum lymph nodes, lung	Elevated ACE	Yes: bloody, bilateral, lymphocytic majority	No	Lung: noncaseating granulomas	Prednisone, marked improvement of the pleural effusion and reduced lymph adenopathy
	55/F	Chronic cough and fever	Hilum and mediastinum lymph nodes, cervical lymph node, pleura	Elevated ACE	Yes: right	No	Lymph node biopsy: granulomatous, pleural nodules: noncaseating granulomas	Methylprednisolone, pleural effusion had disappeared completely
8. Kumagai et al. (71)	64/F	Dyspnea	Skin, hilar and mediastinal lymph nodes, lung, spleen, pleura	Elevated ACE and sIL2R	Yes: bilateral, predominance of lymphocytes	No	Skin and lymph nodes: non-caseous epithelioid granuloma	Prednisolone, completely improvement
9. Fontecha et al. (72)	38/M	Weight loss, pleuritic chest pain, dyspnea	Lung	Elevated ACE	Yes: right, predominantly lymphocytic exudate	No	Lung: necrotizing Epithelioid granulomas	Corticosteroids, Improvement in PE and clinical symptoms
10. Walker et al. (73)	67/F	Dyspnea	Lung, pleura	Elevated ACE	Yes: bilateral, predominantly lymphocytic exudate	No	Lung: non-necrotizing epithelioid granulomas, Pleura: chronic pleuritis with associated granulomata	Prednisolone, azathioprine, Hydroxychloroquine, improvement
11. Enomoto et al. (74)	69/M	Dyspnea	Pleura	Increased lysozyme and calcium	Yes: bilateral, exudative and lymphocytic	No	Miliary nodules on pleural biopsy: epithelioid cell granulomas	Prednisolone, bilateral pleural effusion disappeared
12. Shin et al. (75)	56/F	Shortness of breath	Lung, mediastinal and hilar lymph nodes	Elevated ACE	Yes: left-sided, lymphocyte-predominant transudate	No	Lung: noncaseating granulomas	Prednisone, complete resolution of pleural effusion
13. Emel et al. (76)	56/F	Chest tightness and discomfort, fever, vomiting, coughing	Gastric antrum and lung	Elevated CA-125	Yes: left, pleural thickening	No	Gastric antrum and lung: non-caseating granulomas composed of epithelial and multinucleated giant cells	No follow-up
14. Paone et al. (77)	42/M	Nausea, vomiting, abdominal pain, constipation and fever	Peritoneum, small bowel wall	Anemia	No	Yes, peritonitis, Multiple nodules	Multiple nodules biopsies on bowel wall and peritoneum: lymph histiocytic non-caseating granulomatous inflammation with multinucleated giant cells	Prednisone, a complete response to therapy
15. Hiroaki et al. (78)	46/F	Massive hematemesis, hepatosplenomegaly (5 cm below the right costal margin), erythematous skin lesions	Liver, lymph nodes, skin, heart, stomach, lungs	Elevated ALP and ACE and IL-2, pancytopenia,	No	Yes (because of portal hypertension)	Liver, abdominal lymph nodes, skin lesions, and cardiac muscle and gastric folds biopsies: non-caseating granulomatous inflammation	Prednisolone, no follow-up
16. Jha et al. (79)	65/M	Shortness of breath, fever, weight loss	Lung, pleura, lymph node	Elevated ACE	Yes: right, with pleural thickening, hemorrhagic exudative with high leucocyte count	No	Lung and mediastinal lymph node and pleura biopsies: non-necrotizing granulomatous inflammation	Oral steroids, improved clinic-radiologically
17. Daniel (80)	55/F	Cough, pleuritic chest pain, weight loss	Lung, pleura	Not reported	Yes: right, lymphocytic exudate	No	Lung and pleura: non-caseating granulomas	Prednisone and methotrexate, no recurrence of pleural effusion
18. Abdurrahman et al. (81)	70/F	Fatigue and abdominal pain.	Paraphiliac lymph node	Elevated ALP	Yes: left, exudative flu-id	Yes	Paraphiliac lymph node: non-caseating granulomas	Prednisolone, improvement
19. Lee et al. (82)	55/F	Dry cough and dyspnea, weight loss	Pleura, lung and lymph nodes	Elevated CA-125 and ACE	Yes: left, lymphocytic exudate	NO	Pleura and paratracheal lymph nodes and pulmonary nodule biopsies: non-necrotizing granulomas	Prednisolone, completely improvement in CA-125, ACE and pleural effusion

Above all, sarcoidosis has diverse clinical presentations, and many patients present with atypical symptoms. Thus, it needs to be timely identified by the clinician, and carefully differentiated from other diseases with similar findings, so as to make an accurate diagnosis. In this case, the patient had a poor clinical response to glucocorticoids in the early stage of treatment due to a severe condition and multiorgan involvement. It is worth noting that the patient had improved significantly after 9 months of treatment with corticosteroids, which suggests that multisystemic sarcoidosis requires a long treatment course and a regular follow-up clinic.

## Patient Perspective

The patient believed that our diagnosis and treatment for his disorder were reasonable. Most importantly, his clinical manifestations and laboratory examination results were significantly improved. During the process of glucocorticoid therapy, blood glucose and blood pressure were well controlled, and there was no gastrointestinal discomfort. The only thing that confused him was occasionally depression. The patient indicated that he would continue to comply with our treatment and undergo regular rechecks and feedback.

## Data Availability Statement

All data sets generated for this study are included in the article.

## Ethics Statement

Ethical review and approval was not required for the study on human participants in accordance with the local legislation and institutional requirements. The patients/participants provided their written informed consent to participate in this study. Written informed consent was obtained from the individual(s) for the publication of any potentially identifiable images or data included in this article.

## Author Contributions

XQ and SH drafted the case report. AA, YY, and W-yL performed the biopsy and language modifying. JK, YY, and Q-yW revised the report. All authors contributed to the article and approved the submitted version.

## Funding

This study was supported by the National Clinical Key Specialty Project Foundation (Project Number: 2016YFC 1304103 and 2016YFC 1304500).

## Conflict of Interest

The authors declare that the research was conducted in the absence of any commercial or financial relationships that could be construed as a potential conflict of interest.

## Publisher's Note

All claims expressed in this article are solely those of the authors and do not necessarily represent those of their affiliated organizations, or those of the publisher, the editors and the reviewers. Any product that may be evaluated in this article, or claim that may be made by its manufacturer, is not guaranteed or endorsed by the publisher.
